# Association of Sarcopenia and Visceral Obesity with Clinical Outcomes Among Older Adults with Cardiovascular Disease: A Retrospective Cohort Study

**DOI:** 10.3390/jcm14124191

**Published:** 2025-06-12

**Authors:** Hye-Jin Yoon, Keon-Woo Park, Young-Hoon Seo

**Affiliations:** 1 Dunsan Endocrinology Medical Clinic, Daejeon 35230, Republic of Korea; dldzm0509@naver.com; 2 Department of Internal Medicine, Konyang University Hospital, Daejeon 35365, Republic of Korea; wo7635@gmail.com; 3 Division of Cardiology, Cardio-Cerebrovascular Center, Konyang University Hospital, Daejeon 35365, Republic of Korea

**Keywords:** sarcopenia, visceral obesity, cardiovascular disease, metabolic diseases, heart failure, atherosclerotic cardiovascular disease

## Abstract

**Background/Objectives:** The clinical implications of sarcopenia and visceral obesity in patients with cardiovascular disease (CVD) are poorly understood. We evaluated the impact of sarcopenia and visceral obesity on clinical outcomes among older adults with CVD. **Methods:** This retrospective cohort study included patients aged 65 years and older who had cardiovascular disease and had undergone body composition analysis using dual-energy X-ray absorptiometry (DXA) between November 2021 and October 2022 and been followed through February 2024. Sarcopenia was defined using the 2019 Asian Working Group for Sarcopenia criteria, and visceral obesity was defined using Korean sex-specific visceral adipose tissue area. The primary outcome was a composite of all-cause mortality and major cardiovascular events, including myocardial infarction, stroke, hospitalization for heart failure, and coronary revascularization. This study followed the Strengthening the Reporting of Observational Studies in Epidemiology reporting guidelines. **Results:** A total of 317 patients were included, of whom 118 patients (37.2%) had sarcopenia, 184 (58.0%) had visceral obesity, and 55 (17.4%) had sarcopenic obesity. The prevalence of sarcopenia or visceral obesity was 93.8% in patients with obesity and 69.3% in those without obesity. Sarcopenic obesity showed a mixture of characteristics of two metabolic conditions in terms of demographics and body mass index. Sarcopenia was associated with an increased risk of primary outcomes (hazard ratio [HR], 1.93; 95% CI, 1.02–3.66), with the highest risk observed in patients with sarcopenic obesity (HR, 6.74; 95% CI, 1.81–25.16). **Conclusions:** Sarcopenia was associated with 1.9-fold increased risk of cardiovascular events among older adults with CVD, with a greater than 6-fold increased risk when combined with visceral obesity.

## 1. Introduction

Age-dependent changes in body composition are characterized by a decline in skeletal muscle mass and function and an increase in adipose tissue, particularly in the visceral area [1,2]. The prevalence of sarcopenia and visceral obesity increase with aging, and these two metabolic conditions are closely related and can occur together [3,4]. Although imaging techniques for analyzing body composition are advancing and the concerns regarding the metabolic disease are growing [5,6,7,8], the assessment and management of sarcopenia and visceral obesity remain suboptimal in clinical practice [9,10]. As both sarcopenia and visceral obesity are independently associated with an increased risk of morbidity and mortality, the detection of these metabolic diseases may help prevent adverse cardiovascular events, particularly in high-risk populations [1,11,12].

Cardiovascular disease (CVD) is a major cause of adverse cardiovascular events and mortality, and its prevalence increases with age, similar to sarcopenia and visceral obesity [13,14,15,16]. Age-dependent changes in body composition increase the risk of CVD in older adults regardless of underlying risk factors. Sarcopenia is more prevalent in patients with CVD than in those without and is associated with a greater risk of adverse cardiovascular events [17,18]. Similarly, visceral obesity shares risk factors with CVD and is also an independent risk factor for the development of CVD [11,19,20]. Given that a combination of CVD and metabolic disease may worsen clinical outcomes, the timely screening of metabolic disease should be considered to reduce the morbidity and mortality, especially in older adults with CVD [21].

Little is known about the clinical features and implications of sarcopenia and visceral obesity in patients with established CVD. Most previous studies were cross-sectional, performed outside Asia, involved community-dwelling populations, or did not exclusively analyze older adults [12,18,19,20,21,22,23,24,25]. Furthermore, few studies have used dual-energy X-ray absorptiometry (DXA), which is a non-invasive, inexpensive, safe, and reliable imaging study as compared with computed tomography or magnetic resonance imaging, for measuring body composition [5,6]. Considering that a single DXA examination can assess the decrease in skeletal muscle mass and the accumulation of visceral fat concurrently by analyzing the whole body of the individual, the screening of metabolic disease using DXA in patients with CVD may help identify the presence of sarcopenia and visceral obesity and evaluate their negative synergistic effects. In the present study, we evaluated the clinical features of sarcopenia and visceral obesity among older adults with CVD and investigated their impact on cardiovascular outcomes.

## 2. Methods

### 2.1. Study Design and Population

This retrospective observational cohort study was performed in a single tertiary hospital. We analyzed data of consecutive patients aged 65 years and older who were regularly visiting Konyang University Hospital, Daejeon, Republic of Korea, for heart failure, atherosclerotic cardiovascular disease (ASCVD), or both, and who had undergone body composition analysis using DXA between 1 November 2021 and 31 October 2022. Patients with primary muscle disease, those with cachexia, or those who could not undergo screening for physical performance or muscle strength were ineligible for this study. The diagnosis and classification of heart failure followed the major clinical guidelines [26,27]. ASCVD included stable angina, unstable angina, non-ST-segment elevation myocardial infarction, ST-segment elevation myocardial infarction, percutaneous coronary intervention, stroke, and lower extremity peripheral artery disease [28]. Patients had to be in a stable condition and not require admission or cardiovascular intervention at the time of enrollment. The date of the DXA examination was defined as the index date, and the follow-up data were collected through 29 February 2024. The study followed the Strengthening the Reporting of Observational Studies in Epidemiology reporting guideline [29,30].

### 2.2. Body Composition Analysis and Definitions of Sarcopenia and Visceral Obesity

Body composition was measured using DXA (Lunar Prodigy advance, GE healthcare, Madison, WI, USA) in the research hospital. The fat, muscle, and bone mineral contents of the whole body and each anatomical region, and the mass and area of visceral adipose tissue, were analyzed. Muscle strength was measured using handgrip strength and physical performance was measured using gait speed at index date following the 2019 Asian Working Group for Sarcopenia (AWGS) consensus [31]. Sarcopenia was defined as a low appendicular skeletal muscle mass (ASM) combined with a low muscle strength, low physical performance, or both. Low skeletal muscle mass was defined as height-adjusted ASM less than 7.0 kg/m^2^ in men and less than 5.4 kg/m^2^ in women. Low physical performance was defined as gait speed less than 1.0 m/s for both men and women, and low muscle strength was defined as handgrip strength less than 28 kg in men and less than 18 kg in women. Visceral obesity was defined as visceral adipose tissue area of 134.6 cm^2^ or greater in men and 91.1 cm^2^ or greater in women, using the sex-specific cut-off values obtained from the large-scale Korean population-based study [32].

### 2.3. Clinical Outcomes and Covariates

The occurrence of mortality and cardiovascular events was assessed throughout the study period. The primary outcome was a composite of all-cause mortality and major cardiovascular events comprising myocardial infarction, stroke, hospitalization for heart failure, and coronary revascularization. Follow-up duration was defined as time from the index date to the date of occurrence of the primary outcome, censoring, or the end of the study. Demographics, comorbidities, and the medication use of study participants at the index date were analyzed. Major CVDs other than heart failure or ASCVD were identified. Obesity was defined as a body mass index (BMI, calculated as weight in kilograms divided by height in meters squared) of 25 or greater according to the World Health Organization (WHO) Asia-Pacific definition and Korean Society for the Study of Obesity (KSSO) guideline [33,34]; class 1 obesity was defined as a BMI between 25 and 29.9 and class 2 obesity was defined as a BMI of 30 or greater.

### 2.4. Statistical Analysis

The chi-square test or Fisher’s exact test was used to analyze categorical variables and the independent t-test or Mann–Whitney test was used to analyze continuous variables. Categorical variables were presented as absolute numbers and percentages, and continuous variables were presented as means and standard deviations. All the variables that showed a significant difference at baseline were entered into univariable Cox-proportional hazard analysis to evaluate the hazards ratio (HR) and 95% confidence intervals (CIs), and the variables showing *p* value less than 0.20 in the univariable analysis were entered into the multivariable model. Age, sex, hypertension, diabetes, and chronic kidney disease were considered clinically relevant and entered into multivariable model independently of significance in the univariable analysis. Obesity was prioritized over BMI in cases where both showed a significant difference. Incidence density was presented as the incidence rate per 1000 person-years when appropriate. As the occurrence of myocardial infarction and stroke was low, with incidence rates of 0.3% and 1.3%, respectively, analyses for evaluating the association of sarcopenia and visceral obesity with clinical outcomes were not conducted for these individual outcomes. All statistical analyses were performed using IBM SPSS software (version 28.0, IBM Corp., Armonk, NY, USA) and a *p* value less than 0.05 was considered statistically significant.

## 3. Results

### 3.1. Study Population

A total of 317 patients were included in this study, of whom 143 (45.1%) had heart failure and 236 (74.4%) had ASCVD. The mean age was 75.4 ± 6.6 years, 158 (49.8%) patients were female, and the mean BMI was 24.1 ± 3.6 (Table S1). The prevalence of hypertension, diabetes, dyslipidemia, and obesity was 78.2%, 41.0%, 33.8%, and 35.3%, respectively. In the body composition analysis using DXA, 118 patients (37.2%) had sarcopenia, 184 (58.0%) had visceral obesity, and 55 (17.4%) had both conditions, constituting sarcopenic obesity. The demographics, comorbidities, body composition, medication use, and biochemical characteristics of the study population, arranged according to the presence of sarcopenia and visceral obesity, are given in Table 1. While patients with sarcopenia alone had lowest proportion of female participants (36.5%, n = 23) and the lowest mean BMI (20.8 ± 2.4), those with visceral obesity alone had highest proportion of female participants (61.2%, n = 79) and highest mean BMI (26.8 ± 3.2). When characteristics were compared by the presence of sarcopenia and visceral obesity, the patients with sarcopenia included fewer females and were older than those without sarcopenia (Table S2). These patients were also more likely to have chronic kidney disease and heart failure, and less likely to have obesity and ASCVD. The patients with visceral obesity included more females and were more likely to have hypertension and obesity than those without visceral obesity (Table S3).

### 3.2. Primary Outcome

During the study period, 48 major adverse events occurred in 41 patients (12.9%) (Table S4). There were 11 cases of all-cause mortality and 37 cases of major cardiovascular events. The most common events were hospitalization for heart failure and coronary revascularization (each 16 patients, 5.0%). Myocardial infarction and stroke occurred in 1 and 4 patients, respectively. The mean follow-up duration of overall study population was 17.8 months.

Sarcopenia was associated with an increased risk of a primary outcome before (HR, 2.58; 95% CI, 1.38–4.80) and after adjustment (HR, 1.93; 95% CI, 1.02–3.66), which was mainly driven by the higher all-cause mortality in patients with sarcopenia compared with those without sarcopenia (56.3 vs. 6.9 per 1000 person-years; HR, 8.48; 95% CI, 1.83–39.24) (Table 2 and Table S5). The incidence rate per 1000 person-years for hospitalization for heart failure (57.4 vs. 24.6; HR, 2.25; 95% CI, 0.84–6.05) and coronary revascularization (39.0 vs. 35.7; HR, 1.07; 95% CI, 0.39–2.95) showed no significant difference between patients with and without sarcopenia. Visceral obesity was not linked to the increased risk of primary outcome before (HR, 1.12; 95% CI, 0.60–2.11) and after adjustment (HR, 2.13; 95% CI, 0.99–4.57) (Table 3).

### 3.3. Sarcopenic Obesity

Of 118 patients with sarcopenia, 55 had visceral obesity and 63 did not. The patients with sarcopenic obesity displayed more obesity, lower levels of high-density lipoprotein cholesterol, and more carotid artery disease than those with sarcopenia alone (Table S6). They also had higher trunk-to-total fat ratio, total body fat percentage, total fat mass, and visceral adipose tissue mass. The incidence rate per 1000 person-years of the primary outcome was 245.8 in patients with sarcopenic obesity and 96.5 in those with sarcopenia alone. Sarcopenic obesity, compared with sarcopenia alone, was significantly associated with an increased risk of primary outcomes when unadjusted (HR, 2.46; 95% CI, 1.05–5.75), adjusted for covariates in demographics, comorbidities, biochemical characteristics, and CVD (HR, 3.21; 95% CI, 1.24–8.37), and adjusted for covariates in demographics, comorbidities, biochemical characteristics, CVD, and body composition (HR, 6.74; 95% CI, 1.81–25.16) (Table 4).

### 3.4. Sarcopenia, Visceral Obesity, and Body Mass Index

**Figure 1:**
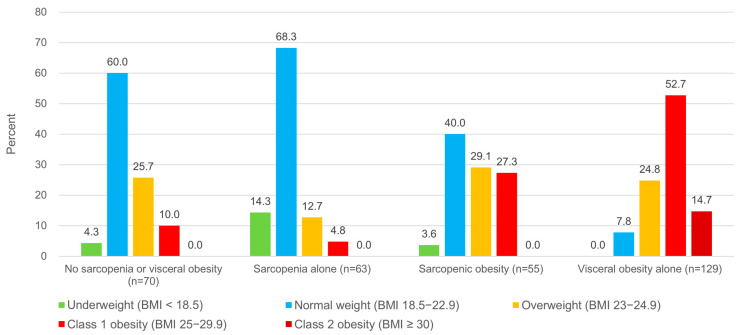
Obesity and class 2 obesity classified using BMI among study population according to presence of sarcopenia and visceral obesity. Abbreviation: BMI, body mass index.

The proportion of underweight, normal weight, overweight, and obesity among study population using the KSSO definition was 4.4%, 36.9%, 23.0%, and 35.7%, respectively. Among 205 patients without obesity, 142 (69.3%) had sarcopenia or visceral obesity, whereas only 63 did not have sarcopenia. Among 112 patients with obesity, 105 (93.8%) had sarcopenia or visceral obesity. Overweight or obese people accounted for 17.5% of patients with sarcopenia alone and 92.2% of those with visceral obesity alone. Among patients with sarcopenic obesity, 56.4% were overweight or obese, showing a mixture of the characteristics of two metabolic conditions (Figure 1). All of the 19 patients who had class 2 obesity had visceral obesity but not sarcopenia. Among participants with class 1 obesity, 95.2% of patients with sarcopenia alone, 32.6% of those with visceral obesity alone, and 72.7% of those with sarcopenic obesity were identified as having sarcopenia or visceral obesity using DXA, and these values correspond to the percentages of patients who could not be screened using BMI. When subjects were limited to class 2 obesity, 85.3% of patients with visceral obesity alone and 100% of patients with sarcopenia with or without visceral obesity were identified as having metabolic conditions using DXA.

## 4. Discussion

In this retrospective cohort study, the authors evaluated the association of sarcopenia and visceral obesity with clinical outcomes among older adults with CVD and demonstrated two important findings. First, sarcopenia and visceral obesity were prevalent in older adults with CVD, and sarcopenia was associated with an increased risk of composite of all-cause mortality and major cardiovascular events, particularly when combined with visceral obesity. These results revealed that the screening of metabolic disease using DXA may be used to identify sarcopenia and visceral obesity and predict clinical outcomes in older adults with CVD. Second, the evaluation of metabolic conditions using BMI alone appeared to be unsuitable, because a majority of older adults who had sarcopenia or visceral obesity were normal weight or overweight, not obese. The prevalence of obesity among patients with sarcopenic obesity, who had the highest risk of cardiovascular events, was only 27.3% in this study. These findings suggest that an appropriate imaging study, such as DXA, may help to detect metabolic diseases that are related to body composition change.

Sarcopenia and visceral obesity are representative metabolic diseases that are asymptomatic and age-dependent. In the previous studies, patients with CVD had more metabolic conditions and were associated with poor prognosis compared with those without CVD. In addition, modifiable and non-modifiable risk factors of metabolic disease were more prevalent in this population. Nevertheless, whether screening metabolic disease in patients with CVD is beneficial remains unclear. We evaluated the presence of sarcopenia and visceral obesity in patients with CVD through a single screening using DXA and demonstrated the impact of these metabolic diseases on clinical outcomes as well as their negative synergistic effects. Given that screening for specific disease should be determined based on efficacy, safety, and cost-effectiveness, our results suggest that DXA would be a feasible imaging method for screening metabolic disease based on its advantages, including that it can be simply and safely performed in clinical settings, compared with other modalities.

Body composition differs by sex and race. Despite the substantial differences in clinical guidelines that assess and define sarcopenia [31,35,36], DXA and bioelectrical impedance analysis have widely been used to estimate body composition [37,38]. The AWGS suggested specific diagnostic criteria for sarcopenia in the Asian population in 2014 and revised it in 2019. During revision, the cut-off value for low muscle strength using handgrip strength in men was changed from 26 kg to 28 kg and for that low physical performance using gait speed was changed from 0.8 m/s to 1.0 m/s. DXA and bioelectrical impedance analysis are used to estimate body composition,

Liang et al. showed that the updated 2019 AWGS criteria were better at predicting mortality due to sarcopenia among community-dwelling Chinese adults compared with the previous 2014 guideline [22]. Therefore, we used the recent 2019 AWGS criteria to define sarcopenia and address its untoward effects. In previous studies, waist circumference; visceral adipose tissue volume, mass, and area; and trunk-to-total fat ratio were used as parameters of visceral obesity because there was no clear consensus regarding its definition. Visceral adipose tissue area, also known as the visceral fat area, showed a good correlation with the risk factors and development of CVD. Sex-specific cut-off values of visceral fat area in Korean adults for predicting incident type 2 diabetes and metabolic syndrome were evaluated by two large-scale studies, presenting similar values [30,39]. Based on these results, we used visceral adipose tissue areas of 134.6 cm^2^ or greater in men and 91.1 cm^2^ or greater in women as the cut-off values of visceral obesity, which were obtained from the largest study.

Another notable aspect of this study is the association of sarcopenia and visceral obesity with BMI. There was a J-shaped association between BMI and all-cause mortality, with the lowest mortality being seen in the BMI range from 23.0 to 24.9 among the Korean population [40]. While the thresholds for the risk of diabetes, myocardial infarction, and stroke were increased from a BMI of 25, the mortality increased from a BMI of 30 [41]. Therefore, KSSO suggests 25 as the cut-off value of BMI for obesity to reduce comorbidities, whereas the WHO suggests 30 to reduce mortality. In our study, the mean BMI of patients with both sarcopenia and visceral obesity, showing the highest incidence of the primary outcome, was 23.5 ± 2.6, which corresponded to the underweight category and the value showing lowest mortality in Korean population. This finding suggests that BMI may appear to be within acceptable range in older adults due to decreases in skeletal muscle and increases in adipose tissue, and that metabolic disease may be underestimated without body composition analysis.

### Strengths and Limitations

This study has several strengths. First, we used most recent Asian-specific consensus for sarcopenia and Korean-specific reference values for visceral obesity to clearly identify two metabolic diseases. We found that both sarcopenia and visceral obesity were prevalent among older adults with CVD and were associated with poor clinical outcomes in this high-risk population. Second, our study was conducted among only patients with CVD and used DXA, a gold-standard method for measuring body composition. Most previous studies were conducted with the general population or using electronic medical records and used other imaging techniques or parameters having insufficient evidence. Third, we found that the use of BMI in older adults was insufficient to detect various metabolic conditions, emphasizing the requirement of imaging study for body composition analysis.

Despite new findings and strengths, our study has some limitations that should be considered. First, our study was conducted in a single center with a small sample size during a short follow-up period. More large-scale and long-term data are needed to overcome this limitation. Second, we used the cut-off values of visceral adipose tissue area that were obtained from a previous study using abdominal computed tomography to define visceral obesity. Although DXA showed good correlation with computed tomography for measuring body composition, the DXA-derived cut-off values of visceral obesity have not been established. Thus, this remains a limitation of our study. Third, there was heterogeneity among the study subjects in terms of CVD subtype. Although this study only included patients with major CVD, there were disparities in the proportion of the subtypes of heart failure and ASCVD. Nevertheless, our study is the first to evaluate sarcopenia and visceral obesity among older adults with CVD using DXA and tailored criteria and validate their impact on cardiovascular outcomes. Fourth, due to the limited number of women included, we could not perform specific analysis in this subgroup.

## 5. Conclusions

Sarcopenia and visceral obesity were prevalent among older adults with CVD, and sarcopenia was associated with an increased risk of composite of all-cause mortality and major cardiovascular events, particularly when combined with visceral obesity. Our study emphasizes that the screening of metabolic disease using DXA in older adults with CVD may help detect the risk factors and improve cardiovascular outcomes. Further studies are needed to evaluate the impact of sarcopenia and visceral obesity on cardiovascular outcomes and to demonstrate the effectiveness of the screening and prevention of these metabolic burdens among older adults with CVD.

## Figures and Tables

**Table 1:** Demographics, comorbidities, body composition, medication use, and biochemical characteristics of study population.

Variables ^a^	No Sarcopenia or Visceral Obesity (n = 70)	Sarcopenia Alone (n = 63)	Visceral Obesity Alone (n = 129)	Sarcopenic Obesity (n = 55)
Demographics				
Age, years	73.7 ± 6.1	77.5 ± 6.0	73.9 ± 6.5	78.5 ± 6.2
Female	30 (42.9)	23 (36.5)	79 (61.2)	26 (47.3)
Height, cm	158.5 ± 10.5	157.0 ± 9.1	157.9 ± 9.6	158.2 ± 9.6
Weight, kg	56.7 ± 8.7	51.6 ± 9.0	67.0 ± 11.3	59.1 ± 10.0
BMI ^b^	22.5 ± 2.1	20.8 ± 2.4	26.8 ± 3.2	23.5 ± 2.6
Comorbidities				
Hypertension	50 (71.4)	44 (69.8)	109 (84.5)	45 (81.8)
Diabetes	30 (42.9)	28 (44.4)	48 (37.2)	24 (43.6)
Dyslipidemia	20 (28.6)	19 (30.2)	51 (39.5)	17 (30.9)
Obesity ^c^	7 (10.0)	3 (4.8)	87 (67.4)	15 (27.3)
Chronic kidney disease	18 (25.7)	21 (33.3)	27 (20.9)	22 (40.0)
Body composition				
Low ASM ^d^	20 (28.6)	63 (100)	6 (4.7)	55 (100)
Low muscle strength ^e^	32 (45.7)	51 (81.0)	71 (55.0)	39 (70.9)
Low physical performance ^f^	23 (32.9)	46 (73.0)	41 (31.8)	51 (92.7)
Trunk-to-total fat ratio, %	52.5 ± 6.2	50.9 ± 7.4	59.4 ± 3.8	60.0 ± 4.4
Body fat percentage, %	25.7 ± 6.1	26.2 ± 6.4	36.1 ± 5.9	36.1 ± 5.1
Total fat mass, g	14,280 ± 3549	13,521 ± 4442	23,997 ± 5302	21,130 ± 3691
VAT mass, g	601 ± 284	607 ± 315	1469 ± 551	1370 ± 436
VAT area, cm^2^	70.7 ± 31.3	72.1 ± 35.2	172.9 ± 59.2	162.3 ± 45.2
Medication use				
ACE inhibitor	7 (10.0)	15 (23.8)	11 (8.5)	7 (12.7)
ARB	24 (34.3)	17 (27.0)	71 (55.0)	26 (47.3)
BB	21 (30.0)	27 (42.9)	58 (45.0)	20 (36.4)
CCB	26 (37.1)	19 (30.2)	73 (56.6)	21 (38.2)
Diuretics	26 (37.1)	34 (54.0)	59 (45.7)	30 (54.5)
Aspirin	25 (35.7)	20 (31.7)	42 (32.6)	14 (25.5)
P2Y12 inhibitor	28 (40.0)	28 (44.4)	53 (41.1)	23 (41.8)
Anticoagulant	21 (30.0)	17 (27.0)	35 (27.1)	20 (36.4)
Statin	58 (82.9)	50 (79.4)	121 (93.8)	48 (87.3)
Biochemical characteristics				
Glycated hemoglobin, %	6.3 ± 1.0	6.4 ± 1.3	6.2 ± 0.7	6.5 ± 1.0
BUN, mg/dL	18.7 ± 7.3	22.0 ± 11.0	18.2 ± 6.1	23.1 ± 12.3
Creatinine, mg/dL	1.0 ± 0.4	1.1 ± 0.9	0.9 ± 0.3	1.3 ± 1.5
GFR, ml/min/1.73 m^2^	75.7 ± 22.3	67.1 ± 22.6	76.2 ± 18.3	61.1 ± 23.0
TC, mg/dL	137.2 ± 37.4	138.1 ± 35.6	143.0 ± 32.1	131.0 ± 29.5
TG, mg/dL	111.7 ± 66.6	107.5 ± 43.9	132.2 ± 52.5	133.4 ± 88.7
HDL, mg/dL	18.3 ± 13.0	48.1 ± 16.7	47.3 ± 11.7	39.8 ± 10.5
LDL, mg/dL	65.2 ± 27.0	68.4 ± 32.3	69.4 ± 27.0	68.3 ± 24.1
Hs-CRP, mg/dL	0.3 ± 0.7	0.5 ± 2.1	0.1 ± 0.3	0.7 ± 2.3

Abbreviations: ACE, angiotensin-converting enzyme; ARB, angiotensin receptor blocker; ASM, appendicular skeletal muscle mass; BB, beta-blocker; BMI, body mass index; BUN, blood urea nitrogen; CCB, calcium channel blocker; GFR, glomerular filtration rate; HDL, high-density lipoprotein; Hs-CRP, high-sensitivity C-reactive protein; LDL, low-density lipoprotein; TC, total cholesterol; TG, triglyceride; VAT, visceral adipose tissue. ^a^ data are presented as number (percentage) of participants unless otherwise indicated. ^b^ calculated as weight in kilograms divided by height in meters squared. ^c^ defined as a BMI of 25 or greater. ^d^ defined as height-adjusted ASM less than 7.0 kg/m^2^ in men and less than 5.4 kg/m^2^ in women. ^e^ defined as handgrip strength less than 28 kg in men and less than 18 kg in women. ^f^ defined as gait speed less than 1.0 m/s.

**Table 2:** Multivariable analysis of association between sarcopenia and primary outcome.

Variables	HR	95% CI	*p* Value
Sarcopenia	1.93	1.02–3.66	0.043
Age	1.07	1.02–1.12	0.008

The final variables entered into multivariable analysis were sarcopenia, age, female, weight, smoking, hypertension, diabetes, obesity, chronic kidney disease, heart failure, and atherosclerotic cardiovascular disease. Abbreviations: CI, confidence interval; HR, hazard ratio.

**Table 3:** Multivariable analysis of association between visceral obesity and primary outcome.

Variables	HR	95% CI	*p* Value
Visceral obesity	2.13	0.99–4.57	0.052
Weight	0.96	0.94–0.99	0.015

The final variables entered into multivariable analysis were visceral obesity, age, female, weight, smoking, hypertension, diabetes, obesity, and chronic kidney disease. Abbreviations: CI, confidence interval; HR, hazard ratio.

**Table 4:** Association between visceral obesity and primary outcomes among patients with sarcopenia.

Participants	Incidence Rate/1000 Person-Years	Unadjusted Model	Model 1	Model 2
		HR (95% CI)	HR (95% CI)	HR (95% CI)
Patients without visceral obesity (n = 63)	96.5	1 (ref)	1 (ref)	1 (ref)
Patients with visceral obesity (n = 55)	245.8	2.46 (1.05–5.75)	3.21 (1.24–8.37)	6.74 (1.81–25.16)

Abbreviations: CI, confidence interval; HR, hazard ratio. Model 1—hazard ratio is adjusted for weight, obesity, triglyceride, high-density lipoprotein cholesterol, and carotid artery disease. Model 2—hazard ratio is adjusted for weight, obesity, triglyceride, high-density lipoprotein cholesterol, carotid artery disease, trunk-to-total fat ratio, total body fat percentage, total fat mass, and visceral adipose tissue mass.

## Data Availability

The original contributions presented in this study are included in the article/Supplementary Material. Further inquiries can be directed to the corresponding author.

## Supplementary Materials

The following supporting information can be downloaded at: https://www.mdpi.com/article/10.3390/jcm14124191/s1.
