# Supplementary material for: Association of Sarcopenia and Visceral Obesity with Clinical Outcomes Among Older Adults with Cardiovascular Disease: A Retrospective Cohort Study
Source: J Clin Med. 2025 Jun 12;14(12):4191. doi: 10.3390/jcm14124191 (PMC12194000; doi:10.3390/jcm14124191)
Supplement: Supplementary file 1 [file jcm-14-04191-s001.zip › jcm-3670669-supplementary.pdf]

Supplementary Materials

TABLE OF CONTENTS

Table S1. Baseline characteristics of study population 2

Table S2. Characteristics of patients with and without sarcopenia 4

Table S3. Characteristics of patients with and without visceral obesity 6

Table S4. Incidence of clinical outcomes 8

Table S5. Univariable analysis of association between sarcopenia and primary outcome 9

Table S6. Incidence rate / 1,000 person-years of clinical outcomes according to presence of sarcopenia ... 10

Table S7. Univariable analysis of association between visceral obesity and primary outcome 11

Table S8. Characteristics of patients with and without visceral obesity among sarcopenic cohort 12

Table S1. Baseline characteristics of study population

Variables[a]	Overall (n=317)
Demographics	
Age, years	75.4±6.6
Female	158 (49.8)
Height, cm	157.9±9.7
Weight, kg	60.3±11.7
Body mass index	24.1±3.6
Smoking	
Current smoker	34 (10.7)
Ex-smoker	107 (33.8)
Never smoker	176 (55.5)
Comorbidities	
Hypertension	248 (78.2)
Diabetes	130 (41.0)
Dyslipidemia	107 (33.8)
Obesity[b]	112 (35.3)
Chronic kidney disease	88 (27.8)
Cardiovascular disease	
Heart failure	143 (45.1)
with reduced EF[c]	16 (11.2)
Pro-BNP, pg/ml	8345±7211
with mildly reduced EF[d]	13 (9.1)
Pro-BNP, pg/ml	4731±6201
with preserved EF[e]	114 (79.7)
Pro-BNP, pg/ml	1565±2144
Atherosclerotic cardiovascular disease	236 (74.4)

Stable angina	102 (32.2)
Unstable angina	11 (3.5)
NSTEMI	33 (10.4)
STEMI	19 (6.0)
Percutaneous coronary intervention	117 (36.9)
Stroke	62 (19.6)
Lower extremity peripheral arterial disease	12 (3.8)
Atrial fibrillation	88 (27.8)
Paroxysmal	22 (6.9)
Persistent	28 (8.8)
Permanent	38 (12.0)
Carotid artery disease	28 (8.8)
Medication use	
Angiotensin-converting enzyme inhibitor	40 (12.6)
Angiotensin receptor blocker	138 (43.5)
Beta blocker	126 (39.7)
Calcium-channel blocker	139 (43.8)
Diuretics	149 (47.0)
Aspirin	101 (31.9)
P2Y12 inhibitor	132 (41.6)
Anticoagulant	93 (29.3)
Statin	277 (87.4)

Abbreviations: BNP, brain natriuretic peptide; EF, ejection fraction; NSTEMI, non-ST-segment elevation myocardial infarction; STEMI, ST-segment elevation myocardial infarction.

[a] Data are presented as number (percentage) of participants unless otherwise indicated. [b] Defined as body mass index of 25 or greater. [c] Defined as EF ≤ 40%. [d] Defined as 40% < EF < 50%. [e] Defined as EF ≥ 50%.

Table S2. Characteristics of patients with and without sarcopenia

Variables[a]	Sarcopenia		
	Yes (n=118)	No (n=199)	*P* value
Demographics			
Age, years	77.9±6.1	73.8±6.4	<0.001
Female	49 (41.5)	109 (54.8)	0.023
Height, cm	157.6±9.3	158.1±9.9	0.316
Weight, kg	55.1±10.2	63.4±11.6	<0.001
Body mass index	22.1±2.8	25.3±3.5	<0.001
Smoking			0.023
Current smoker	11 (9.3)	23 (11.6)	
Ex-smoker	51 (43.2)	56 (28.1)	
Never smoker	56 (47.5)	(120 (68.2)	
Comorbidities			
Hypertension	89 (75.4)	159 (79.9)	0.351
Diabetes	52 (44.1)	78 (39.2)	0.394
Dyslipidemia	36 (30.5)	71 (35.7)	0.347
Obesity[b]	18 (15.3)	94 (47.2)	<0.001
Chronic kidney disease	43 (36.4)	45 (22.6)	0.008
Cardiovascular disease			
Heart failure	67 (56.8)	76 (38.2)	0.001
Atherosclerotic cardiovascular disease	79 (66.9)	157 (78.9)	0.018
Stable angina	33 (38.0)	69 (34.7)	
Unstable angina	4 (3.4)	7 (3.5)	
NSTEMI	13 (11.0)	20 (10.1)	
STEMI	5 (4.2)	14 (7.0)	
Percutaneous coronary intervention	46 (39.0)	71 (35.7)	

Stroke	27 (22.9)	35 (17.6)	
Lower extremity peripheral arterial disease	4 (3.4)	8 (4.0)	
Atrial fibrillation	34 (28.8)	54 (27.1)	0.667
Paroxysmal	10 (8.5)	12 (6.0)	
Persistent	12 (10.2)	16 (8.0)	
Permanent	12 (10.2)	26 (13.1)	
Carotid artery disease	6 (5.1)	22 (11.1)	0.070

Abbreviations: NSTEMI, non-ST-segment elevation myocardial infarction; STEMI, ST-segment elevation myocardial infarction.

[a] Data are presented as number (percentage) of participants unless otherwise indicated. [b] Defined as body mass index of 25 or greater.

Table S3. Characteristics of patients with and without visceral obesity

Variables[a]	Visceral obesity		
	Yes (n=184)	No (n=133)	*P* value
Demographics			
Age, years	75.3±6.7	75.5±6.3	0.755
Female	105 (57.1)	53 (39.8)	0.002
Height, cm	158.0±9.6	157.8±9.9	0.864
Weight, kg	64.7±11.5	54.3±9.2	<0.001
Body mass index	25.8±3.4	21.7±2.4	<0.001
Smoking			0.004
Current smoker	14 (7.6)	20 (15.0)	
Ex-smoker	54 (29.3)	53 (39.8)	
Never smoker	116 (63.0)	60 (45.1)	
Comorbidities			
Hypertension	154 (83.7)	94 (70.7)	0.006
Diabetes	72 (39.1)	58 (43.6)	0.424
Dyslipidemia	68 (37.0)	39 (29.3)	0.156
Obesity[b]	102 (55.4)	10 (7.5)	<0.001
Chronic kidney disease	49 (26.6)	39 (29.3)	0.597
Cardiovascular disease			
Heart failure	83 (45.1)	60 (45.1)	0.999
Atherosclerotic cardiovascular disease	137 (74.5)	99 (74.4)	0.997
Stable angina	58 (31.5)	44 (33.1)	
Unstable angina	5 (2.7)	6 (4.5)	
NSTEMI	19 (10.3)	14 (10.5)	
STEMI	10 (5.4)	9 (6.8)	
Percutaneous coronary intervention	64 (34.8)	53 (39.8)	

Stroke	33 (17.9)	29 (21.8)	
Lower extremity peripheral arterial disease	8 (4.3)	4 (3.0)	
Atrial fibrillation			0.902
Paroxysmal	12 (6.5)	10 (7.5)	
Persistent	18 (9.8)	10 (7.5)	
Permanent	22 (12.0)	16 (12.0)	
Carotid artery disease	20 (10.9)	8 (6.0)	0.133

Abbreviations: NSTEMI, non-ST-segment elevation myocardial infarction; STEMI, ST-segment elevation myocardial infarction.

[a] Data are presented as number (percentage) of participants unless otherwise indicated. [a] Defined as body mass index of 25 or greater.

Table S4. Incidence of clinical outcomes

Variables[a]	Overall (n=317)
Primary outcome[b]	41 (12.9)
All-cause mortality	11 (3.5)
Major cardiovascular events	37 (11.7)
Myocardial infarction	1 (0.3)
Stroke	4 (1.3)
Hospitalization for heart failure	16 (5.0)
Coronary revascularization	16 (5.0)

[a] Data are presented as number (percentage) of participants. [b] The incidence of primary outcome was lower than sum of the incidence of all-cause mortality and major cardiovascular events because the simultaneous occurrence of two or more events was counted as single event.

Table S5. Univariable analysis of association between sarcopenia and primary outcome

Variables	HR	95% CI	*P* value
Sarcopenia	2.58	1.38 – 4.80	0.003
Age	1.09	1.04 – 1.14	<0.001
Female	1.33	0.71 – 2.47	0.374
Weight	0.97	0.97 – 1.00	0.026
Smoking			
Current smoker	1.47	0.60 – 3.61	0.412
Ex-smoker	0.79	0.39 – 1.61	0.511
Obesity[a]	0.57	0.28 – 1.17	0.123
CKD	2.31	1.25 – 4.27	0.006
HF	1.88	1.00 – 3.53	0.049
ASCVD	0.64	0.34 – 1.22	0.175

Abbreviations: ASCVD, atherosclerotic cardiovascular disease; CI, confidence intervals; CKD, chronic kidney disease; HF, heart failure; HR, hazard ratio; NA, not applicable.

[a] Defined as a body mass index of 25 or greater.

Table S6. Incidence rate / 1,000 person-years of clinical outcomes according to presence of sarcopenia

	Incidence rate / 1,000 person-years		HR (95% CI)
Clinical outcomes	Sarcopenia (n=118)	No sarcopenia (n=199)	
Primary outcome	162.2	61.6	2.58 (1.38 – 4.80)
All-cause mortality	56.3	6.9	8.48 (1.83 – 39.24)
Major cardiovascular events			
Hospitalization for heart failure	57.4	24.6	2.25 (0.84 – 6.05)
Coronary revascularization	39.0	35.7	1.07 (0.39 – 2.95)

Abbreviations: CI, confidence intervals; HR, hazard ratio.

As myocardial infarction and stroke occurred in only 1 and 4 patients, respectively, incidence of myocardial infarction and stroke was not compared.

Table S7. Univariable analysis of association between visceral obesity and primary outcome

Variables	HR	95% CI	*P* value
Visceral obesity	1.12	0.60 – 2.11	0.711
Female	1.33	0.71 – 2.47	0.374
Weight	0.97	0.97 – 1.00	0.026
Smoking			
Current smoker	1.47	0.60 – 3.61	0.412
Ex-smoker	0.79	0.39 – 1.61	0.511
Hypertension	1.74	0.73 – 4.14	0.209
Obesity[a]	0.57	0.28 – 1.17	0.123

Abbreviations: CI, confidence intervals; HR, hazard ratio; NA, not applicable.

[a] Defined as a body mass index of 25 or greater.

Table S8. Characteristics of patients with and without visceral obesity among sarcopenic cohort

	Visceral obesity		
**Variables**[a]	**Yes (n=55)**	**No (n=63)**	***P* value**
Demographics			
Age, years	78.5±6.2	77.5±6.0	0.375
Female	26 (47.3)	23 (36.5)	0.236
Height, cm	158.2±9.6	157.0±9.1	0.481
Weight, kg	59.1±10.0	51.6±9.0	<0.001
Body mass index	23.5±2.6	20.8±2.4	<0.001
Smoking			0.518
Current smoker	4 (7.3)	7 (11.1)	
Ex-smoker	22 (40.0)	29 (46.0)	
Never smoker	29 (52.7)	27 (42.9)	
Comorbidities			
Hypertension	45 (81.8)	44 (69.8)	0.132
Diabetes	24 (43.6)	28 (44.4)	0.930
Dyslipidemia	17 (30.9)	19 (30.2)	0.930
Obesity[b]	15 (27.3)	3 (4.8)	<0.001
Chronic kidney disease	22 (40.0)	21 (33.3)	0.453
Biochemical characteristics			
Glycated hemoglobin, %	6.5±1.0	6.4±1.3	0.615
Blood urea nitrogen, mg/dl	23.1±12.3	22.0±11.0	0.596
Creatinine, mg/dl	1.3±1.5	1.1±0.9	0.403
Glomerular filtration rate, ml/min/1.73m^2	61.1±23.0	67.1±22.6	0.158
Total cholesterol, mg/dl	131.0±29.5	138.1±35.6	0.276
Triglyceride, mg/dl	133.4±88.7	107.5±43.9	0.070

High-density lipoprotein cholesterol, mg/dl	39.8±10.5	48.1±16.7	0.002
Low-density lipoprotein cholesterol, mg/dl	68.3±24.1	68.5±32.3	0.981
High sensitivity C-reactive protein, mg/dl	0.7±2.3	0.5±2.1	0.644
Cardiovascular disease			
Heart failure	31 (56.4)	36 (57.1)	0.932
Atherosclerotic cardiovascular disease	37 (67.3)	42 (66.7)	0.944
Stable angina	12 (36.4)	21 (33.3)	
Unstable angina	1 (1.8)	3 (4.8)	
NSTEMI	6 (10.9)	7 (11.1)	
STEMI	1 (1.8)	4 (6.3)	
Percutaneous coronary intervention	19 (34.5)	27 (42.9)	
Stroke	13 (23.6)	14 (22.2)	
Lower extremity peripheral arterial disease	3 (5.5)	1 (1.6)	
Atrial fibrillation			0.508
Paroxysmal	5 (9.1)	7 (7.9)	
Persistent	8 (14.5)	4 (6.3)	
Permanent	5 (9.1)	7 (11.1)	
Carotid artery disease	6 (10.9)	0 (0)	0.009
Body composition			
Trunk-to-total fat ratio, %	60.0±4.4	50.9±7.4	<0.001
Total body fat percentage, %	36.1±5.1	26.2±6.4	<0.001
Total fat mass, g	21130±3691	13521±4442	<0.001
Visceral adipose tissue mass, g	1370±436	607±315	<0.001

Abbreviations: NSTEMI, non-ST-segment elevation myocardial infarction; STEMI, ST-segment elevation myocardial infarction.

[a] Data are presented as number (percentage) of participants unless otherwise indicated. [b] Defined as body mass index of 25 or greater.
